# A Unified Theory of Psychophysical Laws in Auditory Intensity Perception

**DOI:** 10.3389/fpsyg.2020.01459

**Published:** 2020-06-26

**Authors:** Fan-Gang Zeng

**Affiliations:** Center for Hearing Research, Department of Anatomy and Neurobiology–Department of Biomedical Engineering–Department of Cognitive Sciences–Department of Otolaryngology – Head and Neck Surgery, University of California, Irvine, Irvine, CA, United States

**Keywords:** loudness, intensity discrimination, just-noticeable-differences (jnd), Weber’s law, Fechner’s law, Stevens’s law, Zwislocki, auditory

## Abstract

Psychophysical laws quantitatively relate perceptual magnitude to stimulus intensity. While most people have accepted Stevens’s power function as the psychophysical law, few believe in Fechner’s original idea using just-noticeable-differences (jnd) as a constant perceptual unit to educe psychophysical laws. Here I present a unified theory in hearing, starting with a general form of Zwislocki’s loudness function (1965) to derive a general form of Brentano’s law. I will arrive at a general form of the loudness-jnd relationship that unifies previous loudness-jnd theories. Specifically, the “slope,” “proportional-jnd,” and “equal-loudness, equal-jnd” theories, are three additive terms in the new unified theory. I will also show that the unified theory is consistent with empirical data in both acoustic and electric hearing. Without any free parameters, the unified theory uses loudness balance functions to successfully predict the jnd function in a wide range of hearing situations. The situations include loudness recruitment and its jnd functions in sensorineural hearing loss and simultaneous masking, loudness enhancement and the midlevel hump in forward and backward masking, abnormal loudness and jnd functions in cochlear implant subjects. Predictions of these loudness-jnd functions were thought to be questionable at best in simultaneous masking or not possible at all in forward masking. The unified theory and its successful applications suggest that although the specific form of Fechner’s law needs to be revised, his original idea is valid in the wide range of hearing situations discussed here.

## Introduction

Psychophysical laws attempt to relate the amplitude of a physical stimulus to its perceived magnitude, such as loudness as a function of sound pressure or brightness as a function of luminance. The classic approach to uncovering psychophysical laws was advanced by Fechner (1966) in the mid 18th century (original work published in 1860). Fechner assumed that the just-noticeable-difference (jnd), expressed as the Weber fraction (Δ*I*/*I*), where *I* is a standard sound intensity and Δ*I* is the intensity change required for the jnd, produced an equal increment in loudness sensation (Δ*L*). Integrating this equation, namely Δ*L* = Δ*I*/*I*, he produced what is known as Fechner’s law: loudness is a logarithmic function of sound intensity (*L* = *log I*).

Not only was Fechner’s logarithmic law replaced by Stevens’s power law or *L* = *I*^θ^, where θ is a constant (Stevens, 1961), his general approach was also questioned due to failure to integrate the jnd functions of two different sounds to predict their respective loudness functions (Newman, 1933; Miller, 1947). Thus, it was not too surprising that the Fechnerian approach in relating the stimulus jnd to the subjective magnitude was abandoned by some researchers. What was surprising is the grounds on which the Fechnerian approach was abandoned. For example, Stevens (1961) argued that the direct magnitude estimation technique obsolesced intensity discrimination as a measure of the stimulus-sensation relationship. He viewed the discrimination measure as “an engineer talking…the scatter of some dial settings.” In a completely opposing view, Viemeister and Bacon (1988) stated that loudness estimation data were a measure with “probably strong involvement of non-sensory factors, (and) we did not attempt to relate these data to those for intensity discrimination.”

There have been other researchers who continued to advance the Fechnerian approach in searching for a unified theory relating intensity discrimination to the loudness function. Fechner’s original assumption was sometimes referred to as the “slope” theory, because it predicted that the steeper the loudness function, the smaller the jnd or Weber fraction for a constant increment in loudness. This simple slope prediction turned out to be not true at least in cases of loudness recruitment, where cochlear hearing loss or partial masking elevated the hearing threshold but produced abnormally steep loudness growth so that normal loudness was perceived at high sound levels (Fowler, 1937). To account for the failure of Fechner’s slope theory, several researchers proposed a “proportional-jnd” theory, in which the jnd size needed to be normalized by the total jnd number within a stimulus’s dynamic range (Riesz, 1933; Teghtsoonian, 1971; Lim et al., 1977). On the other hand, the “equal-loudness, equal-jnd” theory argued that the jnd had no relation to the slope of the loudness function, but rather was determined by the total loudness (Zwislocki and Jordan, 1986). Despite significant effort in testing these loudness-jnd relationships, no consensus has been reached yet (Houtsma et al., 1980; Hellman et al., 1987; Schlauch and Wier, 1987; Rankovic et al., 1988; Johnson et al., 1993; Stillman et al., 1993; Schlauch et al., 1995; Allen and Neely, 1997; Hellman and Hellman, 2001).

Here I present a unified theory, starting with a general form of Zwislocki’s (1965) loudness function to derive a general form of Brentano’s law, and I will arrive at a general form of the loudness-jnd relationship that unifies previous loudness-jnd theories. Specifically, I find that the previous “slope,” “proportional-jnd,” and “equal-loudness, equal-jnd” theories, are three additive terms in the new unified theory. I also show that the new theory is capable of predicting loudness and jnd data across a wide range of hearing situations, including sensorineural hearing loss, simultaneous masking, forward masking, and electric hearing.

## Derivation of a Unified Theory

### Derivation of a General Form of Brentano’s or Ekman’s Law

I start with the general form of a loudness function proposed by Zwislocki (1965; Eq. 212):

(1) $$L=k\left[{\left(I+cI_{0}\right)}^{\theta }-{\left(cI_{0}\right)}^{\theta }\right]$$

where *I*_0_ is the detection threshold for a particular type of sound, *c* represents an internal noise scaling factor, and *k* is a constant.

Generality and symmetry are the two reasons for choosing Zwislocki’s loudness function. First, at high intensities (*I* ≫ *I*_*o*_), Zwislocki’s function can be simplified as Stevens’s power law, namely, *L* = *kI*^θ^. At low intensities, Zwislocki made an implicit but important assumption to account for loudness recruitment near threshold: The slope (θ) of the loudness function does not increase as initially thought (Fowler, 1937), instead the loudness at threshold is increased. Setting *I* = *I*_*o*_ in Eq. (1), the loudness at threshold, or *L*_*o*_ = *k*[(*I_*o*_* + *cI*_*o*_)^θ^ − (*cI*_*o*_)^θ^] = *k* [(*1/c* + *1*)^θ^ − 1)] (*cI*_*o*_)^θ^ ∼ *k* [θ (*1/c*)*^1–θ^*] (*I*_*o*_)^θ^, is directly proportional to the threshold and “must be greater than zero (Zwislocki, 1965; p. 87).” Mathematically, the loudness at threshold is infinite when the internal noise is zero (*c* = 0), and vice versa. This is a fundamental argument for why the brain has or needs internal noise because infinite loudness is clearly biologically unacceptable. Zwislocki’s internal noise concept was also expanded to form the basis for treating loudness recruitment as “softness imperception” (Buus and Florentine, 2002) and tinnitus as “additive central noise” (Zeng, 2013). In the interest of simplicity, I define loudness at threshold as: *L*_*o*_ = *k*(*cI*_*o*_)^θ^ (or *c* = 0.125 for θ = 0.27).

Second, the mathematical symmetry can be shown by differentiating Eq. (1):

(2) $$\frac{\Delta L}{\Delta I}=\theta k{\left(I+cI_{0}\right)}^{\theta -1}=\theta k\frac{{\left(I+cI_{0}\right)}^{\theta }}{I+cI_{0}}$$

Adding and subtracting the same component in the above equation, I obtain:

(3) $$\frac{\Delta L}{\Delta I}=\theta k\frac{{\left(I+cI_{0}\right)}^{\theta }-{\left(cI_{0}\right)}^{\theta }+{\left(cI_{0}\right)}^{\theta }}{I+cI_{0}}=\theta \frac{L+L_{0}}{I+cI_{0}}$$

Rewriting the above equation, I obtain the general form of Brentano’s law or Ekman’s law, namely, $\frac{\Delta L}{L}=\frac{\Delta I}{I}$, (see Stevens, 1961, for discussion of these laws):

(4) $$\frac{\Delta L}{L+L_{0}}=\theta \frac{\Delta I}{I+cI_{0}}$$

Equation (4) is mathematically symmetrical and balanced, having a general form of Weber’s law including a threshold-correction term in both the sensation domain (*L*_*o*_) and the stimulus domain (c*I*_*o*_).

To the first-order approximation, Weber’s law in the stimulus domain has been “replicated in hundreds of studies across all sensory modalities and many animal species over the last two centuries (Pardo-Vazquez et al., 2019).” In auditory intensity discrimination, the Weber fraction is constant for broadband noise but decreases slightly with increasing intensity, resulting in a “near miss” to Weber’s law (McGill and Goldberg, 1968). Therefore, Eq. (4) can be written as:

(5) $$\frac{\Delta L}{L+L_{0}}=wI^{\alpha }$$

where *w* and α are both constants, with α = 0 indicating perfect conformity to Weber’s law.

According to the “proportional-jnd” theory (Lim et al., 1977), the constant *w* is inversely proportional to the number of jnds (*N*) within the stimulus dynamic range. In other words, *w* = 1/*N*, which can be considered as a scaling factor to account for the fact that different subjects or different types of stimuli may have a different number of discriminable steps within their respective dynamic range (e.g., a normal-hearing listener has 100 steps but a cochlear-implant user has only 10), but they all have similar loudness growth from soft at the threshold to uncomfortably loud at the upper limit of the range. The “proportional-jnd” theory states that 10 jnd steps in the normal-hearing listener would produce the same amount of loudness change as one jnd step in the cochlear-implant user. Although the “proportional-jnd” theory did not assume or require any specific jnd-loudness function, Lim et al. (1977) hinted that Brentano’s law “is nearly the correct one” (see footnote 7 on p. 1264 in Lim et al., 1977). In this case, a relative change in loudness is inversely proportional to the number of jnds with an intensity correction term, whose origin will be considered in section “Discussion”:

(6) $$\frac{\Delta L}{L+L_{0}}=\frac{1}{N}I^{\alpha }$$

### Prediction of the jnd Function From the Loudness Balance Function

Suppose that the loudness function for a tone in quiet is: *L* = *f(I)*, and that the loudness balance function between the tone in quiet and the tone in masking has been obtained: *I* = *g(I_*m*_).* By definition, at *I* = *g(I_*m*_)*, loudness is balanced so that the loudness function can be derived for a partially masked tone:

(7) $$L_{m}=L=f\left(I\right)=f\left[g\left(I_{m}\right)\right]$$

Differentiating the above equation to obtain:

(8) $$\frac{\Delta L_{m}}{\Delta I_{m}}=f^{\prime }\left(I\right)g^{\prime }\left(I_{m}\right)=\frac{\Delta L}{\Delta I}g^{\prime }\left(I_{m}\right)$$

Rewrite the above equation:

(9) $$\Delta I_{m}=\Delta I\frac{1}{g^{\prime }\left(I_{m}\right)}\frac{\Delta L_{m}}{\Delta L}$$

Replace Δ*L*_*m*_ and Δ*L* with Eq. (6) to obtain:

(10) $$\Delta I_{m}=\Delta I\frac{1}{g^{\prime }\left(I_{m}\right)}\frac{N}{N_{m}}\frac{I_{m}^{\alpha }}{I^{\alpha }}\frac{L_{m}+L_{mo}}{L+L_{o}}$$

To predict the jnd in the form of the Weber fraction at the same intensity, that is, *I*_*m*_ = *I* so that one can cancel out the intensity correction term ($I_{m}^{\alpha }/I^{\alpha }$) and divide the above equation by (*I*):

(11) $$\frac{\Delta I_{m}}{I}=\frac{\Delta I}{I}\frac{1}{g^{\prime }\left(I_{m}\right)}\frac{N}{N_{m}}\frac{L_{m}+L_{mo}}{L+L_{o}}$$

Taking a logarithmic transformation, one can calculate the jnd in terms of the Weber fraction in dB (WFdB):

(12) $$\begin{array}{c} {\mathrm{WF}}_{m}\mathrm{dB}(I)=WFdB\left(I\right)-10\log g\prime \left(I_{m}\right)+10\text{log}\frac{N}{N_{m}}\\ \;+10\text{log}\frac{L_{m}+L_{mo}}{L+L_{o}} \end{array}$$

where *WF_*m*_dB(I)* = 10log(Δ*I_*m*_/I*), which is the log Weber fraction for a masked tone and *WFdB(I)* = 10log(Δ*I/I*), which is the log Weber fraction for a tone in quiet.

Equation (12) indicates that, if *WFdB(I)* is known at a given intensity (*I*), then one can predict *WF_*m*_dB(I)* at the same intensity from three additional measures: (1) the local slope of the loudness balance function [*g’*(*I*_*m*_)], (2) a scaling factor (*N/N_*m*_*), and (3) the local loudness ratio between the masked tone and the tone in quiet [(*L_*m* +_ L_*mo*_)/(L* + *L*_*o*_)]. Interestingly, in theory, there is no need to know explicitly the detection threshold, nor the exact form of loudness growth or intensity discrimination function for the tone in quiet.

I consider Eq. (12) as a unified theory of psychophysical laws in auditory intensity perception because the last three terms in the equation contain the three previous theories that attempted to relate the jnd function to the loudness function. The 10log*g’*(*I*_*m*_) term represents Fechner’s original “slope” theory; the 10log(*N/N_*m*_*) term represents Riesz’s “proportional-jnd” theory; and the final term represents Zwislocki’s “equal-loudness, equal-jnd” theory.

## Validation of the Unified Theory

### Prediction of the jnd Functions in Simultaneous Masking

Simultaneous masking not only elevates a pure tone’s threshold but also affects its loudness perception, similar to loudness recruitment in sensorineural hearing loss. Both loudness balance and intensity discrimination functions have been measured in the same group of listeners for pure tones in quiet and in simultaneous noise maskers (Houtsma et al., 1980; Rankovic et al., 1988; Schlauch et al., 1995).

**FIGURE 1:**
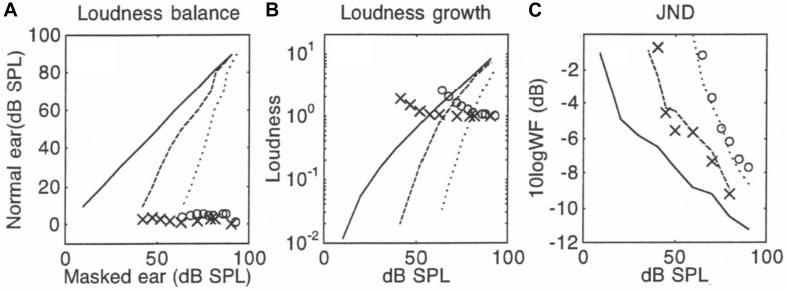
Predictions in simultaneous masking, with data (lines) being from Schlauch et al. (1995). Panel **(A)** shows loudness balance functions between a tone in quiet (*y*-axis) and a tone in noise (*x*-axis): The solid line represents the control condition where the same tone was balanced between the two ears in quiet, the dashed line represents the balance function for a tone being masked by a 15-dB SPL/Hz broadband noise, and the dotted line represents the loudness balance function for a tone by a 40-dB SPL/Hz noise. The symbols represent slope values for the balance function. The slope values use the same scale as the balance function from 0 to 100, except the slopes are unitless. Panel **(B)** shows derived loudness growth functions. The symbols represent loudness ratio values between quiet and masked tones and tones in quiet. Panel **(C)** shows the measured jnd functions (lines) and predicted jnd values (symbols).

Here, I use the Schlauch et al. (1995) data to predict the masked jnd from the quiet jnd because Schlauch et al. (1995) had the most complete set of data. Figure 1 illustrates the relative contributions of the three special terms in Eq. (12) to predictions of the jnd data in simultaneous masking. Figure 1A shows three loudness balance functions: the solid line represents a hypothetical condition where the same tone is perfectly balanced in loudness (i.e., 1:1 ratio) between two ears in quiet, the dashed line represents the measured balance function for a masked tone in a 15-SPL/Hz broadband noise and the dotted line for a masked tone in a 40-dB SPL/Hz broadband noise (from Figure 3 in Schlauch et al., 1995). An interpolation of the loudness balance function is then differentiated to derive the slopes as a function of intensity (X’s represent the 15 dB SPL/Hz masking and O’s represent the 40 dB SPL/Hz masking condition). Figure 1B shows the loudness growth function for a 1000-Hz tone in quiet (solid line) based on Zwislocki’s model [Eq. (1), using *k* = 3.1; θ = 0.27; *c* = 2.5; *I*_*o*_ = 10*^–^*^12^ W/m^2^ or 0 dB SPL], as well as the two masked loudness growth functions obtained by applying the loudness balance functions in Figure 1A to the loudness growth function in quiet. The X’s and O’s represent the loudness ratio between the corresponding quiet and masking conditions. Figure 1C shows measured jnd functions in quiet (solid line), 15-dB masking (dashed line), and 40-dB masking (dotted line). The X’s and O’s represent the predicted jnd values in the above two masking conditions based on Eq. (12). In addition to using the slope values in Figure 1A and loudness ratio values in Figure 1B, Eq. (12) uses a normalization factor of 4 dB and 8 dB for the 15-dB and 40-dB masking conditions, respectively. The 4-dB and 8-dB normalization factor was estimated from the both the dynamic range and the jnd values (Nelson et al., 1996; see their Figure 9), with the quiet condition having 2.5 times and 6.3 times more jnd steps than the 15-dB and 40-dB masking condition, respectively. There was no free parameter in this prediction. In terms of relative contributions to the successful prediction, the “equal-loudness, equal-jnd” theory was essential to the prediction of the overall trend (the same downward pattern in Figures 1B,C), while the slope theory (the relatively flat pattern of the X and O symbols in Figure 1A) behaved similarly to the proportional jnd theory as a constant to shift the predicted function up or down.

### Prediction of the jnd Function in Forward Masking

Loudness and its jnd functions of a stimulus can also be affected by forward and backward masking. Loudness is enhanced and intensity discrimination is degraded in forward and backward masking, particularly at middle intensities (Zeng et al., 1991; Plack and Viemeister, 1992; Zeng and Turner, 1992). Although an early attempt to relate the “midlevel hump” (the jnd function) to loudness enhancement was not successful (Zeng, 1994), Oberfeld (2008) found a significant correlation between the elevated jnd and enhanced loudness when a wide range of masker-to-signal level differences was tested.

**FIGURE 2:**
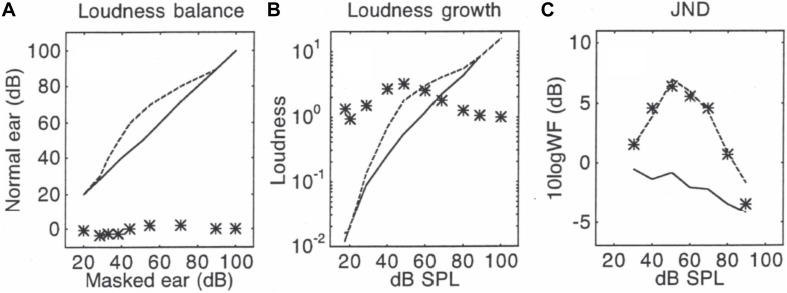
Predictions in forward masking, with data (lines) from Zeng (1994). Panel **(A)** shows loudness balance functions between a tone in quiet (*y*-axis) and a tone in forward masking (*x*-axis): The solid line represents the control condition where the same tone was balanced between the two ears in quiet, while the dashed line represents the balance function for a tone in forward masking. The * symbols represent slope values for the balance function, which uses the same scale as the balance function from 0 to 100, except the slopes are unitless. Panel **(B)** shows derived loudness growth functions. The symbols represent loudness ratio values between the masked tone and the tone in quiet. Panel **(C)** shows the measured jnd functions (lines) and predicted jnd values (symbols).

Using the same processing steps as in Figures 1, 2 shows the loudness balance function between a 25-ms tone in quiet and in the presence of a 90-dB SPL, 100-ms forward masker (Figure 2A), the derived loudness growth function (Figure 2B), and the measured as well as predicted jnd functions in quiet and masking (Figure 2C). The slope theory (Figure 2A) predicted that forward masking would produce smaller than normal jnds for standard levels below 50 dB SPL but larger jnds for levels above 50 dB SPL. The “equal-loudness, equal-jnd” theory (Figure 2B) predicted the midlevel hump jnd function due to enhanced loudness in forward masking. A 7-dB normalization factor, or five times less jnd steps in forward masking, was used in the final successful prediction (Figure 2C) that combined all three special theories in Eq. (12). The similar pattern between Figures 2B,C is generally consistent with the observed correlation between enhanced loudness and elevated jnd (Oberfeld, 2008), but the quantitative prediction needs further investigation. It would be also interesting to know if the present unified theory could predict a similar jnd function observed for brief high-frequency tones under notched noise conditions (Carlyon and Moore, 1984). Oxenham and Moore (1995) hinted such a possibility by proposing “a new theory [that] explain[s] the severe departure from Weber’s law in terms of both the variance… and the loudness of partially masked signals.”

### Predictions of the jnd Functions in Electric Hearing

**FIGURE 3:**
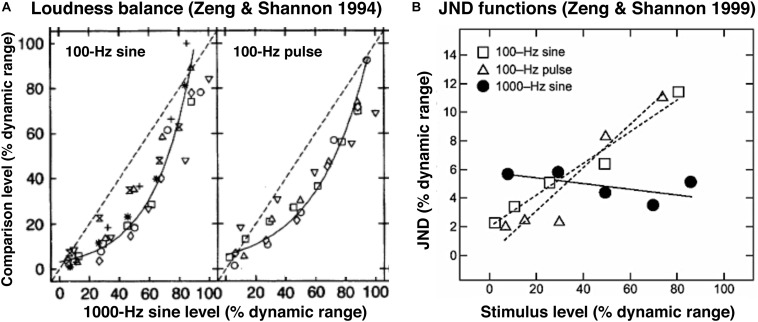
Loudness balance **(A)** and JND functions **(B)** in cochlear implant users. **(A)** Loudness balance functions were obtained between 100-Hz sine or 100-Hz pulse and 1000-Hz sine electric stimuli, adapted from Figures 2D,E in Zeng and Shannon (1994). Reprinted with permission from AAAS. Symbols represent individual data and the solid line represents a logarithmic balance function. The dashed line represents a linear balance function, which clearly was not the true. **(B)** JND data (symbols) and predicted functions (lines) using the same stimuli from the same subjects in **(A)**, adapted from Figure 4 in Zeng and Shannon (1999). Reprinted with permission from Wolters Kluwer Health.

In electric hearing where hair cells are missing and the auditory nerve fibers are directly stimulated by electric currents, loudness generally has a narrow dynamic range of 10–20 dB (Zeng and Galvin, 1999). Zeng and Shannon (1994) found that, in cochlear implant users, loudness grows as a traditional power function of electric current for stimulus frequencies lower than 300 Hz, but as an exponential function for stimulus frequencies higher than 300 Hz. These two different loudness growth functions would produce a logarithmic loudness balance function between low- and high-frequency electric stimuli. Figure 3A shows, indeed, such a logarithmic balance function (solid lines) between a 100-Hz stimulus (sinusoid or pulse amplitude on *y*-axis) and a 1000-Hz sinusoid (*x*-axis).

(13) $$E_{1000Hz}=\theta \log E_{100Hz}$$

where θ is the slope of the logarithmic loudness balance function. Differentiating the above equation to derive the following JND function between the high- and low-frequency electric stimuli:

(14) $$\Delta E_{1000Hz}=\theta \frac{\Delta E_{100Hz}}{E_{100Hz}}$$

Zeng and Shannon (1999) measured jnds of these stimuli in the same implant subjects (symbols in Figure 3B) and found that not only did this jnd function hold but more importantly the jnd function was nearly constant (the solid line in Figure 3B). Given the same power loudness growth function for the 100-Hz electric stimuli, it is not surprising that their Weber fraction was also constant. But why was the absolute difference (Δ*E*_1000 *Hz*_) constant for the 1000-Hz stimulus? Zeng and Shannon (1999) showed that this constant absolute difference was a result of the exponential loudness growth function.

(15) $$L_{1000Hz}=\exp\left(E_{1000Hz}\right)$$

Differentiating the above equation to obtain:

(16) $$\frac{\Delta L_{1000Hz}}{\Delta E_{1000Hz}}=\exp\left(E_{1000Hz}\right)=L_{1000Hz}$$

Rewriting the above equation to obtain:

(17) $$\frac{\Delta L_{1000Hz}}{L_{1000Hz}}=\Delta E_{1000Hz}$$

Equation (17) means that Brentano’s ratio is also constant in electric stimulation. The only difference between Eqs. (17) and (4) is that (17) does not contain a threshold term, probably due to a lack of spontaneous neural activity in the deafened ear (Kiang and Moxon, 1972).

## Discussion

None of the individual components in the present unified theory is new. Previous studies have proposed these individual theories and evaluated them separately (e.g., Zwislocki and Jordan, 1986; Hellman and Hellman, 1990, 2001; Schlauch, 1994; Schlauch et al., 1995; Allen and Neely, 1997). The present study is novel in two respects. First, the present study integrates the previously disconnected individual components through a unified theoretical framework, namely, the general form of Brentano’s law in Eq. (4). Second, the present study offers a new formula, namely, Eq. (12), which specifically combines these individual terms to successfully predict the loudness and jnd relationships in simultaneous and forward masking, as well as in cochlear implant users. The present unified theory and its successful applications suggest that although Weber’s law needs to be replaced by the general form of Brentano’s law, Fechner’s original idea using jnds to derive psychophysical laws is valid at least in the wide range of hearing situations examined here.

**FIGURE 4:**
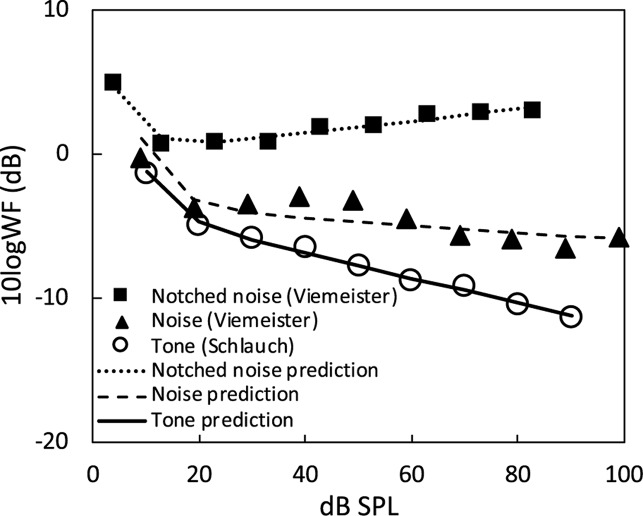
Prediction of JND for noise and tone stimuli. The JND data for a broadband noise (solid triangles) and the same noise in a notched-noise background (solid squares) were from Viemeister (1983; the same symbols in his Figure 1) and the 1000-Hz tone JND data (open circles) were from Schlauch et al. (1995; circles in their Figure 2 bottom-right panel). The dashed line represents prediction of the noise JND function, the dotted line represents the noise in a notched-noise background, and the solid line represents the tone JND function.

The general form of Brentano’s law can be used to examine how close the actual jnd data follow Weber’s law and its potential mechanisms by combining Eqs. (4) and (5):

(18) $$\frac{\Delta L}{L+L_{0}}=\theta \frac{\Delta I}{I+cI_{0}}=wI^{\alpha }or\frac{\Delta I}{I+cI_{0}}=w^{\prime }I^{\alpha }$$

where both *w’*(= *w*/θ) and α are free parameters to be estimated, with α = 0 indicating perfect conformity to Weber’s law. Figure 4 shows the jnd data and the model estimation for a 1-kHz tone (Schlauch et al., 1995), 8-kHz broadband noise (6–14 kHz) and the same noise in a notched noise background (Viemeister, 1983). All three sets of data can be modeled by a two-stage function, with a steep first stage (∼10–20 dB SPL) reflecting the threshold influence and a shallower second stage (∼20–100 dB SPL) with its slope being α in Eq. (16). All three sets of data follow the near-miss to Weber’s law (McGill and Goldberg, 1968), with α being −0.09 for the tone, −0.03 for the noise, and 0.04 for the noise in a notched noise background. The near-miss ranges from −9% to 4% and has an average of 3% for the three stimuli considered here.

To provide a solution to the near-miss to Weber’s law, McGill and Goldberg adopted a Poisson-like process, in which the loudness mean (*L*) and its variance (σ^2^) are equal, where σ is the standard deviation. To achieve 75% correct detection in a jnd task, the signal detection theory requires: $d^{\prime }=\frac{\Delta L}{\sigma }=\frac{\Delta L}{L^{0.5}}=1$ (Green and Swets, 1966). Replacing Δ*L* = *L*^0.5^ in Eq. (19) to produce:

(19) $$\frac{\Delta L}{L+L_{0}}=\frac{L^{0.5}}{L+L_{0}}\propto L^{-0.5}\propto {\left(I^{0.27}\right)}^{-0.5}\propto I^{-0.14}$$

Compared with the −0.14 slope predicted by the Poisson-like process, the estimated slope was is 5% off for the tone, 11% off for the noise and 18% off for the noise in a notched-noise background. As an overcorrection, McGill and Goldberg’s solution has created a much greater difference (average = 11%) than the original problem, i.e., the near-miss (average = 3%) to Weber’s law. Alternatively, the use of spread of excitation cue is the more likely mechanism underlying the near-miss to Weber’s law (Florentine and Buus, 1981; Viemeister, 1983), but a quantitative treatment of its predictive accuracy is still lacking. At least as a first-order approximation, Weber’s law holds for sound intensity discrimination.

While it is challenging, the search for a unified psychophysical law has continued to attract attention, especially on its biological basis (e.g., Shepard, 1987; Nieder and Miller, 2003; Dehaene et al., 2008; Dzhafarov and Colonius, 2011; Teghtsoonian, 2012; Pardo-Vazquez et al., 2019). In an influential paper, which drew 30 open peer commentaries, Krueger (1989) attempted to reconcile Fechner and Stevens by proposing a unified psychophysical law, in which (1) “each jnd has the same subjective magnitude for a given modality,” (2) “subjective magnitude increases as approximately a power function of physical magnitude,” and (3) “subjective magnitude depends primarily on peripheral sensory processes, that is, no non-linear central transformations occur.” With regard to (1), Krueger preferred Δ*S* or in the present term Δ*L* = *c* (constant) for the law of parsimony, but was willing to accept Δ*L*/*L* = *c* (Brentano’s Law) or even Δ*L*/*L* = *L*^−0.5^ (McGill and Goldberg’s Poisson process). The present study favors Brentano’s Law with a threshold correction factor. The second point was the primary concern of Kruger’s unified law, in which not only did he attempt to reconcile the different ways to measure sensation magnitude (e.g., magnitude estimate versus categorical rating), but also derive the subjective magnitude function from the jnd data. He explicitly examined the “proportional-jnd theory” (p. 260), implicitly discussed the “slope” theory (his Table 1 on p. 261), but probably didn’t know about the “equal-loudness, equal-jnd” theory, letting alone consider them as three independent factors that collectively contribute to the jnd-loudness function (the present study). Kruger’s third point treating the brain as a linear device is wrong, because not only does the present study (B3) show that electric stimulation of the auditory nerve, which bypasses the auditory hair cells, produces an exponential loudness function in cochlear implant users, but more importantly many studies on neuroplasticity have found abnormally increased gain in the brain in response to reduced input in the periphery (e.g., Qiu et al., 2000; Norena, 2011; Chambers et al., 2016).

## Data Availability Statement

The datasets generated for this study are available on request to the corresponding author.

## Ethics Statement

Ethical approval was not required for this study as the human data were from previously published studies. The patients/participants provided their written informed consent to participate in this study.

## Author Contributions

F-GZ is solely responsible for the work presented here.

## Conflict of Interest

The author declares that the research was conducted in the absence of any commercial or financial relationships that could be construed as a potential conflict of interest.

## References

[B1] Allen J. B. Neely S. T. (1997). Modeling the relation between the intensity just-noticeable difference and loudness for pure tones and wideband noise. *J. Acoust. Soc. Am.* 102 3628–3646. 10.1121/1.420150

[B2] Buus S. Florentine M. (2002). Growth of loudness in listeners with cochlear hearing losses: recruitment reconsidered. *J. Assoc. Res. Otolaryngol.* 3 120–139. 10.1007/s101620010084 12162363 PMC3202402

[B3] Carlyon R. P. Moore B. C. (1984). Intensity discrimination: a severe departure from Weber’s law. *J. Acoust. Soc. Am.* 76 1369–1376. 10.1121/1.391453 6512099

[B4] Chambers A. R. Resnik J. Yuan Y. Whitton J. P. Edge A. S. Liberman M. C. (2016). Central gain restores auditory processing following near-complete cochlear denervation. *Neuron* 89 867–879. 10.1016/j.neuron.2015.12.041 26833137 PMC4760846

[B5] Dehaene S. Izard V. Spelke E. Pica P. (2008). Log or linear? Distinct intuitions of the number scale in Western and Amazonian indigene cultures. *Science* 320 1217–1220. 10.1126/science.1156540 18511690 PMC2610411

[B6] Dzhafarov E. N. Colonius H. (2011). The fechnerian idea. *Am. J. Psychol.* 124 127–140. 10.5406/amerjpsyc.124.2.0127 21834399

[B7] Fechner G. T. (1966). *Elemente der Psychophysik [Elements of Psychophysics].* New York, NY: Holt, Rinehart and Winston, Inc.

[B8] Florentine M. Buus S. (1981). An excitation-pattern model for intensity discrimination. *J. Acoust. Soc. Am.* 70 1646–1654. 10.1121/1.387219

[B9] Fowler E. P. (1937). Measuring the sensation of loudness a new approach to the physiology of hearing and the functional and differential diagnostic tests. *Arch. Otolaryngol.* 26 514–521. 10.1001/archotol.1937.00650020568002

[B10] Green D. M. Swets J. A. (1966). *Signal Detection Theory and Psychophysics.* New York, NY: Wiley.

[B11] Hellman R. Scharf B. Teghtsoonian M. Teghtsoonian R. (1987). On the relation between the growth of loudness and the discrimination of intensity for pure tones. *J. Acoust. Soc. Am.* 82 448–453. 10.1121/1.395445 3624649

[B12] Hellman W. S. Hellman R. P. (1990). Intensity discrimination as the driving force for loudness. Application to pure tones in quiet. *J. Acoust. Soc. Am.* 87 1255–1265. 10.1121/1.398801 2324392

[B13] Hellman W. S. Hellman R. P. (2001). Revisiting relations between loudness and intensity discrimination. *J. Acoust. Soc. Am.* 109(5 Pt 1), 2098–2102. 10.1121/1.1366373 11386561

[B14] Houtsma A. J. Durlach N. I. Braida L. D. (1980). Intensity perception XI. Experimental results on the relation of intensity resolution to loudness matching. *J. Acoust. Soc. Am.* 68 807–813. 10.1121/1.384819 7419815

[B15] Johnson J. H. Turner C. W. Zwislocki J. J. Margolis R. H. (1993). Just noticeable differences for intensity and their relation to loudness. *J. Acoust. Soc. Am.* 93 983–991. 10.1121/1.405404 8445133

[B16] Kiang N. Y. Moxon E. C. (1972). Physiological considerations in artificial stimulation of the inner ear. *Ann. Otol. Rhinol. Laryngol.* 81 714–730. 10.1177/000348947208100513 4651114

[B17] Krueger L. E. (1989). Reconciling fechner and stevens - toward a unified psychophysical law. *Behav. Brain Sci.* 12 251–267. 10.1017/S0140525x0004855x

[B18] Lim J. S. Rabinowtiz W. M. Braida L. D. Durlach N. I. (1977). Intensity perception. VIII. Loudness comparisons between different types of stimuli. *J. Acoust. Soc. Am.* 62 1256–1267. 10.1121/1.381641 915119

[B19] McGill W. J. Goldberg J. (1968). A study of the near-miss involving Weber’s law and pure-tone intensity discrimination. *Percept. Psychophys.* 4 105–109. 10.3758/bf03209518

[B20] Miller G. (1947). Sensitivity to changes in the intensity of white noise and its relation to masking and loudness. *J. Acoust. Soc. Am.* 19 609–619. 10.1121/1.1916528

[B21] Nelson D. A. Schmitz J. L. Donaldson G. S. Viemeister N. F. Javel E. (1996). Intensity discrimination as a function of stimulus level with electric stimulation. *J. Acoust. Soc. Am.* 100(4 Pt 1), 2393–2414. 10.1121/1.417949 8865646

[B22] Newman E. (1933). The validity of the just noticeable difference as a unit of psychological magnitude. *Trans. Kansas Acad. Sci.* 36 172–175. 10.2307/3625353

[B23] Nieder A. Miller E. K. (2003). Coding of cognitive magnitude: compressed scaling of numerical information in the primate prefrontal cortex. *Neuron* 37 149–157. 10.1016/s0896-6273(02)01144-1143 12526780

[B24] Norena A. J. (2011). An integrative model of tinnitus based on a central gain controlling neural sensitivity. *Neurosci. Biobehav. Rev.* 35 1089–1109. 10.1016/j.neubiorev.2010.11.003 21094182

[B25] Oberfeld D. (2008). The mid-difference hump in forward-masked intensity discrimination. *J. Acoust. Soc. Am.* 123 1571–1581. 10.1121/1.2837284 18345845

[B26] Oxenham A. J. Moore B. C. J. (1995). Overshoot and the severe departure from webers law. *J. Acoust. Soc. Am.* 97 2442–2453. 10.1121/1.411965

[B27] Pardo-Vazquez J. L. Castineiras-de Saa J. R. Valente M. Damiao I. Costa T. Vicente M. I. (2019). The mechanistic foundation of Weber’s law. *Nat. Neurosci.* 22 1493–1502. 10.1038/s41593-019-0439-7 31406366

[B28] Plack C. J. Viemeister N. F. (1992). Intensity discrimination under backward masking. *J. Acoust. Soc. Am.* 92 3097–3101. 10.1121/1.404205 1474224

[B29] Qiu C. Salvi R. Ding D. Burkard R. (2000). Inner hair cell loss leads to enhanced response amplitudes in auditory cortex of unanesthetized chinchillas: evidence for increased system gain. *Hear. Res.* 139 153–171. 10.1016/s0378-5955(99)00171-9 10601720

[B30] Rankovic C. M. Viemeister N. F. Fantini D. A. Cheesman M. F. Uchiyama C. L. (1988). The relation between loudness and intensity difference limens for tones in quiet and noise backgrounds. *J. Acoust. Soc. Am.* 84 150–155. 10.1121/1.396981 3411042

[B31] Riesz R. (1933). The relationship between loudness and the minimum perceptible increment of intensity. *J. Acoust. Soc. Am.* 4 211–216. 10.1121/1.1915601

[B32] Schlauch R. S. (1994). Intensity resolution and loudness in high-pass noise. *J. Acoust. Soc. Am.* 95 2171–2179. 10.1121/1.410017 8201113

[B33] Schlauch R. S. Harvey S. Lanthier N. (1995). Intensity resolution and loudness in broadband noise. *J. Acoust. Soc. Am.* 98 1895–1902. 10.1121/1.413375 7593914

[B34] Schlauch R. S. Wier C. C. (1987). A method for relating loudness-matching and intensity-discrimination data. *J. Speech Hear. Res.* 30 13–20. 10.1044/jshr.3001.13 3560891

[B35] Shepard R. N. (1987). Toward a Universal law of generalization for psychological science. *Science* 237 1317–1323. 10.1126/science.3629243 3629243

[B36] Stevens S. S. (1961). To honor fechner and repeal his law: a power function, not a log function, describes the operating characteristic of a sensory system. *Science* 133 80–86. 10.1126/science.133.3446.80 17769332

[B37] Stillman J. A. Zwislocki J. J. Zhang M. Cefaratti L. K. (1993). Intensity just-noticeable differences at equal-loudness levels in normal and pathological ears. *J. Acoust. Soc. Am.* 93 425–434. 10.1121/1.405622 8423259

[B38] Teghtsoonian R. (1971). On the exponents in Stevens’ law and the constant in Ekman’s law. *Psychol. Rev.* 78 71–80. 10.1037/h0030300 5545194

[B39] Teghtsoonian R. (2012). The standard model for perceived magnitude: a framework for (almost) everything known about it. *Am. J. Psychol.* 125 165–174. 10.5406/amerjpsyc.125.2.0165 22774680

[B40] Viemeister N. F. (1983). Auditory intensity discrimination at high frequencies in the presence of noise. *Science* 221 1206–1208. 10.1126/science.6612337 6612337

[B41] Viemeister N. F. Bacon S. P. (1988). Intensity discrimination, increment detection, and magnitude estimation for 1-kHz tones. *J. Acoust. Soc. Am.* 84 172–178. 10.1121/1.396961 3411045

[B42] Zeng F. G. (1994). Loudness growth in forward masking: relation to intensity discrimination. *J. Acoust. Soc. Am.* 96 2127–2132. 10.1121/1.410154 7963026

[B43] Zeng F. G. (2013). An active loudness model suggesting tinnitus as increased central noise and hyperacusis as increased nonlinear gain. *Hear. Res.* 295 172–179. 10.1016/j.heares.2012.05.009 22641191 PMC3593089

[B44] Zeng F. G. Galvin J. J. III (1999). Amplitude mapping and phoneme recognition in cochlear implant listeners. *Ear Hear.* 20 60–74. 10.1097/00003446-199902000-00006 10037066

[B45] Zeng F. G. Shannon R. V. (1994). Loudness-coding mechanisms inferred from electric stimulation of the human auditory system. *Science* 264 564–566. 10.1126/science.8160013 8160013

[B46] Zeng F. G. Shannon R. V. (1999). Psychophysical laws revealed by electric hearing. *Neuroreport* 10 1931–1935. 10.1097/00001756-199906230-00025 10501535

[B47] Zeng F. G. Turner C. W. (1992). Intensity discrimination in forward masking. *J. Acoust. Soc. Am.* 92(2 Pt 1), 782–787. 10.1121/1.403947 1506532

[B48] Zeng F. G. Turner C. W. Relkin E. M. (1991). Recovery from prior stimulation. II: effects upon intensity discrimination. *Hear. Res.* 55 223–230. 10.1016/0378-5955(91)90107-k 1757290

[B49] Zwislocki J. (1965). “Analysis of some auditory characteristics,” in *Handbook of Mathematical Psychology*, eds Luce R. Bush R. R. Galanter E. (New York, NY: John Wiley and Sons, Inc), 79–97.

[B50] Zwislocki J. J. Jordan H. N. (1986). On the relations of intensity jnd’s to loudness and neural noise. *J. Acoust. Soc. Am.* 79 772–780. 10.1121/1.393467 3958318

